# Neuroblastoma and nephroblastoma: an overview and comparison

**DOI:** 10.1186/1470-7330-14-S1-O15

**Published:** 2014-10-09

**Authors:** Maureen Dumba, Noorulhuda Jawad, Kieran McHugh

**Affiliations:** 1 Department of Radiology, Great Ormond Street Hospital for Children, Great Ormond Street, London, WC1N 3JH, UK

## 

Neuroblastoma (NBL) is the most common extra-cranial tumour in childhood [1] and commonly presents as an abdominal mass. Nephroblastoma, also more commonly known as a Wilms’ tumour, is the commonest renal tumour in childhood and also typically presents as abdominal pathology. The natural histories and typical clinical courses of these tumours are very different, thus early distinction is important. Both occur in early childhood, with Wilms’ having a slightly older peak incidence at between 3 – 4 years. Histologically, they are different diseases with NBLs arising from primordial neural crest cells [1] and Wilms’ being undifferentiated mesodermal tumours [2]. The heterogeneity of NBLs and their biological characteristics mean the prognosis is highly variable; tumour stage, patient age, tumour oncogenes and DNA content are all known to be implicated [3]. Wilms’ have two distinct histopathological types, favorable and unfavorable, with differing outcomes. NBLs are most commonly located within the adrenal gland, so typically present as palpable abdominal masses causing pain and distension [1,4]. Well described paraneoplastic syndromes include opsomyoclonus and excessive vasoactive intestinal peptide (VIP) production [1]. Metastatic disease is common on presentation [1]. Wilms’ usually present as a large, painless abdominal mass with few constitutional symptoms [2]. On ultrasound (US), NBLs are solid, heterogeneous masses with calcification and are rarely cystic [4]. With Wilms’, US also evaluates whether the mass is intra- or extra-renal, solid or cystic, and for the presence of vascular invasion [2]. MRI effectively assesses the extent of primary NBL disease, being superior to CT in assessing metastatic marrow disease, chest wall invasion and spinal canal involvement. On CT, NBLs are poorly marginated, heterogeneous masses that can cross the midline and enter adjacent body cavities. A key-defining feature is calcification, but this can have a variable appearance [4]. NBLs tend to encase and displace structures rather than invade them. Over 90% are MIBG-sensitive, but for primaries that are not, ⁹⁹mTc-diphosphonate bone scintigraphy is currently recommended to look for metastatic bony disease [1,3]. MRI is also useful at Wilms’ diagnosis, with contrast-enhanced sequences clearly demonstrating the ‘claw’ of normal renal tissue around the tumour. Tumours return low signal on T1W, with variable/high signal intensity on T2W. The non-cystic components typically restrict on diffusion sequences. In contrast to NBL, vessels are displaced rather than encased and vascular invasion occurs in approximately 5-10% of cases [4]. NBL staging has evolved with the simpler International Neuroblastoma Risk Group (INRG) system [3,5]. Imaging significantly contributes to this system. To enable consistent reporting, imaging defined risk factors have been identified by the INRG [3]. Unlike with NBL, chest CT is routinely done for Wilms’ staging. Management strategies for NBL include surgery, chemotherapy and radiotherapy, with additional myeloablative therapy and recently also immunotherapy for high-risk disease. A unilateral Wilms’ tumour is treated with nephrectomy and chemotherapy. With bilateral disease, pre-operative chemotherapy is vital as each kidney is staged separately, but the surgical approach is to preserve normal renal parenchyma.

## References

[B1] Maris JM Hogarty MD Bagatell R Cohn SL Neuroblastoma Lancet 2007 369 9579 2106 2120 10.1016/S0140-6736(07)60983-0 17586306

[B2] Davidoff AM Wilms’ tumor Advances in Pediatrics 2012 59 1 247 267 10.1016/j.yapd.2012.04.001 22789581 PMC3589819

[B3] Brisse HJ McCarville MB Granata C Krug KB Wootton-Gorges SL Kanegawa K Giammarile F Schmidt M Shulkin BL Matthay KK Lewington VJ Sarnacki S Hero B Kaneko M London WB Pearson WB Cohn SL Monclair T International Neuroblastoma Risk Group Project. Guidelines for imaging and staging of neuroblastic tumors: consensus report from the International Neuroblastoma Risk Group Project Radiology 2011 261 1 243 57 10.1148/radiol.11101352 21586679

[B4] McHugh K Renal and adrenal tumours in children Cancer Imaging 2007 7 41 51 10.1102/1470-7330.2007.0007 17339140 PMC1828369

[B5] McCarville MB Imaging neuroblastoma: what the radiologist needs to know Cancer Imaging 2011 11 1A S44 S47 10.1102/1470-7330.2011.9008 22187096 PMC3266565

